# Pathway-based analysis for genome-wide association study data of bipolar disorder provides new insights for genetic study

**DOI:** 10.1007/s13238-015-0201-1

**Published:** 2015-09-11

**Authors:** Suhua Chang, Jinglu Wang, Kunlin Zhang, Jing Wang

**Affiliations:** Key Laboratory of Mental Health, Institute of Psychology, Chinese Academy of Sciences, Beijing, 100101 China; University of Chinese Academy of Sciences, Beijing, 100049 China

**Dear Editor,**

Bipolar disorder (BD) is an episodic recurrent pathological mood disturbance with estimated heritability ranging from 80%–85% (Barnett and Smoller, 2009). Although many studies have indicated that BD is a polygenic disease influenced by many genes with small effect, the pathogenesis of BD is still not well understood. According to the catalog of published genome-wide association studies (GWAS) (Welter et al., 2014), there have been 31 BD GWASs (including bipolar disorder related symptoms and combined GWAS with other related disorders) till 01/15/2015, including the largest BD GWAS from the Psychiatric Genomics Consortium (PGC) (Sklar et al., 2011). But these GWAS analyses mainly focused on single SNP/gene and only identified a number of significant SNPs that account for a small proportion of the genetic variants. To detect the association between pathway and trait for further GWAS data interpretation and mechanism study, pathway-based analysis (PBA) has been introduced to analyze GWAS data (Wang et al., 2010).

Comparing with traditional genetic association analyses, PBA can detect the additive effects of multiple minor genes. It has been proved to be a feasible solution to interpret GWAS result and promote the analytical level from SNP/gene to pathway. In recent years, a number of PBA methods have been developed whose null hypothesis being tested can be broadly classified as ‘self-contained’ versus ‘competitive’ based on whether comparisons were made between genes in a specific pathway and non-associated genes or other genes in the genome. Additionally, the input data of these statistical methods can be broadly classified into two types: SNP *P-*values and raw genotype (Wang et al., 2010).

Among the several published PBA studies for BD (as collected in BDgene (Chang et al., 2013)), the majority of the data sources focused on the Wellcome Trust Case Control Consortium (WTCCC) data (Wellcome Trust Case Control 2007), which was published several years ago. The large scale of PGC data has been used in two PBA studies (Duncan et al., 2014; Yu et al., 2014a), but they are both hypothesis-based and only focus on one type of pathway instead of all pathways. In addition, using more than one PBA tools to get the consistent result has become more popular (Duncan et al., 2014) although most of the early studies only used one. By now, the results of published PBA studies are still of low repeatability and need further replication. Hence, it is necessary to further analyze the large scale of GWAS data from PGC by using PBA methods to get more reliable disease related pathways.

In this study, we analyzed six BD GWAS datasets from PGC (Sklar et al., 2011) by merging them into two groups according to the platform. The same quality control (QC) process was conducted for both groups of data by using PLINK (see supplementary material for detail). After QC, the two groups of datasets contained 4568 cases and 6255 controls in total, and the numbers of overlapped SNPs were 276,592 in Group A and 477,624 in Group B separately (as shown in Table S1). We used three tools (*i*-GSEA4GWAS v2 (Zhang et al., 2014), SNP Ration Test (SRT) (O’Dushlaine et al., 2009) and GenGen (Wang et al., 2007)) for our analysis. The brief introduction and parameter selection about these three tools were described in supplementary material.

The pathway-based analysis results for two groups of GWAS datasets by three PBA tools were summarized in Table 1. By inputting SNP *P*-value list, *i*-GSEA4GWAS v2 generated 31 candidate pathways for Group A and 31 candidate pathways for Group B, which shared 12 pathways between Group A and Group B. The calculation of gene-set enrichment score was similar between *i*-GSEA4GWAS v2 and GenGen, but GenGen used genotype data as input and permutation was conducted at phenotype level. GenGen got 133 candidate pathways from Group B, but none from Group A. Furthermore, SRT provided 10 candidate pathways from Group A and 5 candidate pathways from Group B, and there was no overlapped pathway between Group A and Group B. Comparison among different PBA tools for the same GWAS dataset was conducted. For Group A, *i*-GSEA4GWAS v2 shared four pathways with SRT; no shared pathway for either other pairs of tools and three tools. For Group B, *i*-GSEA4GWAS v2 shared 27 pathways and two pathways with GenGen and SRT separately; GenGen and SRT shared three pathways, in which, two pathways were shared by three tools.

**Table 1:** Comparison result for different groups of GWAS datasets and PBA tools

	Group A	Group B	Overlapped among Group A and Group B
*i*-GSEA4GWAS v2	31	31	12
Overlapped in GenGen	0	27	
Overlapped in SRT	4	2	
Overlapped in three tools	0	2	
GenGen	0	133	0
Overlapped in SRT	0	3	
Overlapped in *i*-GSEA4GWAS v2	0	27	
Overlapped in three tools	0	2	
SRT	10	5	0
Overlapped in GenGen	0	3	
Overlapped in *i*-GSEA4GWAS v2	4	2	
Overlapped in three tools	0	2	

As a result, 33 unique pathways were obtained from the shared pathways by GWAS datasets or PBA tools. The list of pathways and supported datasets and tools were shown in Table 2. Among these 33 pathways, one pathway “TGF beta signaling pathway” was validated by one PBA tool, two pathways (“Oocyte meiosis” and “Ubiquitin mediated proteolysis”) were validated by all three PBA tools, other pathways were validated by two PBA tools. Moreover, six pathways had FDR < 0.05 in Group A dataset and six pathways had FDR < 0.05 in Group B dataset. Especially, ‘Retinol metabolism’ and ‘Metabolism of xenobiotics by cytochrome P450’ had FDR < 0.05 in both Group A and Group B datasets, which showed great possibility of correlation with BD.

**Table 2:** Validated pathways by two groups of GWAS datasets or at least two PBA tools

KEGG ID	Pathway	Supported datasets	Supported tools^a^		
			*i*-GSEA4GWAS v2 (FDR)	GenGen (FDR)	SRT (Empirical P)
hsa00983	Drug metabolism-other enzymes	Group A-Group B	0.001 (A), 0.0602 (B)	0.025 (B)	
hsa00830	Retinol metabolism	Group A-Group B	0.0025 (A), 0.0438 (B)	0.021 (B)	
hsa00140	Steroid hormone biosynthesis	Group A-Group B	0.003 (A), 0.1941 (B)	0.043 (B)	
hsa00860	Porphyrin and chlorophyll metabolism	Group A-Group B	0.003 (A), 0.0658 (B)	0.034 (B)	
hsa00980	Metabolism of xenobiotics by cytochrome p450	Group A-Group B	0.0077 (A), 0.0357 (B)	0.004 (B)	
hsa00982	Drug metabolism - cytochrome p450	Group A-Group B	0.0264 (A), 0.1413 (B)	0.019 (B)	
hsa04722	Neurotrophin signaling pathway	Group A-Group B	0.0586 (A), 0.1478 (B)	0 (B)	
hsa04914	Progesterone-mediated oocyte maturation	Group A-Group B	0.0632 (A), 0.0665 (B)	0.003 (B)	
hsa04350	TGF beta signaling pathway	Group A-Group B	0.1426 (A), 0.1456 (B)		
hsa04960	Aldosterone-regulated sodium reabsorption	Group A-Group B	0.1501 (A), 0.1383 (B)	0.249 (B)	
hsa05214	Glioma	Group A-Group B	0.1507 (A), 0.1466 (B)	0.108 (B)	
hsa05340	Primary immunodeficiency	Group A-Group B	0.2111 (A), 0.2086 (B)		0.011 (A)
hsa05217	Basal cell carcinoma	Group B	0.2146 (B)	0 (B)	
hsa04114	Oocyte meiosis	Group B	0.043 (B)	0 (B)	0.007 (B)
hsa04512	ECM-receptor interaction	Group B	0.1643 (B)	0.001 (B)	
hsa00510	N-glycan biosynthesis	Group B	0.152 (B)	0.001 (B)	
hsa05412	Arrhythmogenic right ventricular cardiomyopathy (ARVC)	Group B	0.2058 (B)	0.002 (B)	
hsa00601	Glycosphingolipid biosynthesis lacto and neolacto series	Group B	0.1406 (B)	0.002 (B)	
hsa04540	Gap junction	Group B	0.166 (B)	0.003 (B)	
hsa04720	Long-term potentiation	Group B	0.0482 (B)	0.003 (B)	
hsa04120	Ubiquitin mediated proteolysis	Group B	0.019 (B)	0.002 (B)	0.021 (B)
hsa04115	p53 signaling pathway	Group B	0.109 (B)	0.003 (B)	
hsa04260	Cardiac muscle contraction	Group B	0.0456 (B)	0.007 (B)	
hsa02010	ABC transporters	Group B	0.1515 (B)	0.008 (B)	
hsa00040	Pentose and glucuronate interconversions	Group B	0.2056 (B)	0.039 (B)	
hsa05219	Bladder cancer	Group B	0.15 (B)	0.042 (B)	
hsa03420	Nucleotide excision repair	Group B	0.1332 (B)	0.046 (B)	
hsa04730	Long-term depression	Group B	0.1464 (B)	0.058 (B)	
hsa00591	Linoleic acid metabolism	Group B	0.1444 (B)	0.111 (B)	
hsa04110	Cell cycle	Group B		0.001 (B)	0.042 (B)
hsa04142	Lysosome	Group A	0.2291 (A)		0.008 (A)
hsa04070	Phosphatidylinositol signaling system	Group A	0.201 (A)		0.043 (A)
hsa05221	Acute myeloid leukemia	Group A	0.2093 (A)		0.044 (A)

^a^ (A) Denotes the FDR or Empirical P in Group A, (B) denotes the FDR or Empirical P in Group B. FDR < 0.05 in *i*-GSEA4GWAS v2 are bold

Our analysis also validated several pathways reported by previous PBA studies. For example, phosphatidylinositol signaling system and p53 signaling pathway were identified as risk pathways by Chen et al. using a risk-scoring measurement to fuse SNPs and pathways (Chen et al., 2009); N-Glycan biosynthesis, ABC transporters and cell cycle were identified in (Peng et al., 2010) by using hypergeometric test; Neurotrophin signaling pathway has not been reported by PBA of BD yet, but it has been reported by network analysis paper for schizophrenia (Yu et al., 2014b), and neurotrophic signaling cascades has played an important role in the pathophysiology and treatment of bipolar disorder (Shaltiel et al., 2007).

Besides the pathways validated by other PBA papers, we also identified several novel pathways, which have not been reported to be associated with bipolar disorder by PBA study but have been validated in other literature and deserve further attention, including ‘Ubiquitin mediated proteolysis’ and ‘Oocyte meiosis’, which were validated by three PBA tools in our analysis and also statistical significant in Group A dataset (FDR < 0.05); ‘Retinol metabolism’ and ‘Metabolism of xenobiotics by cytochrome P450’, which were identified in both two groups with significant association with BD (*P*-value < 0.05, FDR < 0.05). Evidence for the association of these pathways with bipolar disorder and other related psychiatric disorders from literature was summarized in the supplementary material.

In summary, compared with previous PBA studies on BD, our analysis includes more samples and uses genotype data to analyze all KEGG pathways. By using multiple GWAS datasets and PBA tools, our analysis could provide better interpretation for the GWASs of BD to further identify disease-related mechanism. The pathways we identified would provide new insights for the genetic and mechanism study of BD. In particular, the novel pathways we first identified such as ‘Ubiquitin mediated proteolysis’, ‘Oocyte meiosis’ and ‘Metabolism of xenobiotics by cytochrome P450’ would provide new perspectives to the following studies. These pathways need and also deserve further validation and replication to explore the pathogenic mechanism of BD.

## Electronic supplementary material

Supplementary material 1 (PDF 178 kb)
