# Supplementary material for: Pathway-based analysis for genome-wide association study data of bipolar disorder provides new insights for genetic study
Source: Protein Cell. 2015 Sep 11;6(12):912–5. doi: 10.1007/s13238-015-0201-1 (PMC4656210; doi:10.1007/s13238-015-0201-1)
Supplement: Supplementary file 1 — Supplementary material 1 (PDF 178 kb) [file 13238_2015_201_MOESM1_ESM.pdf]

Pathway-based analysis for genome-wide association study data of bipolar disorder provides new insights for genetic study

Suhua Chang[1 #], Jinglu Wang[1,2#], Kunlin Zhang[1], Jing Wang[1*]

[1] Key Laboratory of Mental Health, Institute of Psychology, Chinese Academy of Sciences, Beijing 100101, China

[2] University of Chinese Academy of Sciences, Beijing, 100049, China

Supplementary material

Table S1 Six BD GWAS datasets used for PBA.

Group name	GWAS dataset	Ethnicity	Sample size (cases/ controls)	Number of genotyped SNPs	Chip platform
Group A	Systematic Treatment Enhancement Program for Bipolar Disorder (STEP1)	European-American	922/645	316,562	Affymetrix 500K
Group A	University College London (UCL)	European	457/495	329,185	Affymetrix 500K
Group A	Wellcome Trust Case-Control Consortium (WTCCC)	European	1868/2938	465,422	Affymetrix 500K
	After QC		3229/4076	276,592	Affymetrix 500K
Group B	Trinity College Dublin	European	150/797	616,289	Affymetrix 6.0
Group B	Thematically Organized Psychosis (TOP) Study	European	203/349	596,891	Affymetrix 6.0
Group B	Genetic Association Information Network European-American ancestry (GAIN_EA)	European-American	1001/1034	729,454	Affymetrix 6.0
	After QC		1339/2179	477,624	Affymetrix 6.0

Materials and methods

GWAS datasets

The six BD GWAS datasets for this analysis (as shown in Stable 1) include Systematic Treatment Enhancement Program for Bipolar Disorder (STEP1), University College London (UCL), Trinity College Dublin (Irish) and Thematically Organized Psychosis (TOP) Study (Norwegian) from NIMH Repository and Genomics Resource (Distribution 8.0), GWAS for BD from Wellcome Trust Case-Control Consortium (WTCCC), and Genetic Association Information Network European-American ancestry (GAIN_EA, Accession No. phs000017.v3.p1) from dbGaP. The same quality control (QC) process was conducted for both groups of data by using PLINK (Purcell et al., 2007): (i) exclude SNPs with missing rate >0.05 or minor allele frequency <0.05 (before sample removal below), (ii) exclude individuals with missing rate >0.05 or heterozygosity rate outside ± 3 SD (standard derivation), (iii) calculate identity by descent (IBD) after pruning SNPs using r^2=0.2 as threshold, and then exclude duplicated or related individuals with IBD>0.185, and (iv) exclude SNPs with significant differences in case and control call rates ($P<10^{-5}$) or minor allele frequency >0.05 or Hardy-Weinberg equilibrium $P<10^{-6}$. Principal component estimation was done with the same collection of SNPs for IBD calculation using EIGENSTRAT (Price et al., 2006). We estimated the first 20 principle components and evaluated their impact on the genome-wide test statistics using λ as (Ripke et al., 2011). Based on this, we used the top five principle components with the smallest λ together with the study indicator variables as associated covariates for logistic regression test to get *P*-values of SNPs.

***i*-GSEA4GWAS v2**

i-GSEA4GWAS v2 (http://gsea4gwasv2.psych.ac.cn/) is a free web server as an open platform for researchers to analyze GWAS data (Zhang et al., 2014). Unlike the conventional gene set enrichment analysis (GSEA) (Subramanian et al., 2005) with a heavy dependence on genotype data, this approach uses SNP label permutation instead of phenotype label permutation to calculate *P*-values, which makes it easily available for most published GWAS investigations. Besides, based on the enrichment score (ES), *i*-GSEA4GWAS v2 uses the significance proportion based enrichment score (SPES), which emphasizes the total significance coming from high proportion of significant genes to improve sensitivity. The SNP *P*-value list from association analysis was used as the input of *i*-GSEA4GWAS v2. We mapped the SNPs in the 5 kb upstream and downstream of gene to genes. The pathway dataset, which was served as the search space of our PBA, was from Kyoto Encyclopaedia of Genes and Genomes (KEGG) (Ogata et al., 1999). Only the pathways with size (number of genes involved) more than 20 and less than 200 were used in our PBA for BD. Other input parameters were default. Those pathways with false-discovery rate (FDR) < 0.25 were kept as candidate pathways associated with BD.

SNP Ratio Test

The SNP ratio test (SRT) (O'Dushlaine et al., 2009) compares the proportion of significant SNPs to all SNPs within genes that are part of a pathway and computes an empirical *P*-value based on comparisons to ratios in datasets where the assignment of case/control status has been randomized. The input is the genotype data of GWAS in PLINK binary format. The same KEGG pathways as we used in *i*-GSEA4GWAS v2 were

used for pathway searching. Mapping rule from SNPs to genes was also 5 kb upstream and downstream. Standard association analyses were performed for both the original data and 1000 randomized phenotype datasets. Finally, a list containing the empirical *P*-value for the statistically significant enrichment of GWAS associated SNPs within each KEGG pathway was generated. Those pathways with *P*-value < 0.05 were kept as candidate pathways associated with BD.

GenGen

GenGen is a modified GSEA algorithm to perform pathway-based analysis of GWAS data. It takes the maximum statistic for all SNPs near a gene to represent the significance of the gene and uses a permutation-based approach that shuffles the phenotype labels of cases and controls to adjust for multiple testing (Wang et al., 2007). The input file for GenGen is genotype data. The same KEGG pathways and SNP -> gene mapping rule were used as the other two tools. According to our data size, 1000 permutation cycles were used to adjust for multiple-hypothesis testing. Those pathways with FDR< 0.25 were selected as final results.

Literature evidence for new identified pathways

Besides the pathways validated by other PBA papers, we also identified several novel pathways, which have not been reported to be associated with bipolar disorder by PBA study but have been validated in other literature and deserve further attention, including ‘Ubiquitin mediated proteolysis’ and ‘Oocyte meiosis’, which were validated by three PBA tools in our analysis and also statistical significant in Group A dataset (FDR

< 0.05); 'Retinol metabolism' and 'Metabolism of xenobiotics by cytochrome P450', which were identified in both two groups with significant association with BD (P-value $<$ 0.05, FDR < 0.05).

Protein ubiquitination plays an important role in eukaryotic cellular processes; and it mainly functions as a signal for 26S proteasome dependent protein degradation. Now, proteolysis by the ubiquitin-proteasome pathway (UPP) is widely recognized as a molecular mechanism controlling myriad normal functions in the nervous system (Hegde and Upadhya, 2011). This pathway has been identified as a canonical pathway associated with several neuropsychiatric and brain disorders, including Alzheimer's (Lam et al., 2000), Parkinson's (Shimura et al., 2001), bipolar disorder (Bousman et al., 2010), ethanol-induced disorders (Donohue, 2002; Hegde and Upadhya, 2011) and schizophrenia (Middleton et al., 2002; Altar et al., 2005). Recently, Maria et al. have reported the abnormalities of ubiquitination system in schizophrenia by using gene expression analysis (Rubio et al., 2013). The evidence supported the association of 'Ubiquitin mediated proteolysis' with bipolar disorder. Meiosis is a specialized type of cell division, which reduces the chromosome number by half. Although there is no direct evidence showing that oocyte meiosis is related to BD, a study has pointed out that ubiquitin-proteasome pathway plays important roles in oocyte meiosis resumption, spindle assembly, polar body emission, and pronuclear formation, probably by regulating cyclin B1 degradation and MAPK/p90rsk phosphorylation (Huo et al., 2004). Among these, 'MAPK signaling pathway' is reported by two PBA studies for BD (Chen et al., 2009; Peng et al., 2010). Moreover, there is a report that the aggregation of abnormally phosphorylated Tau proteins, which is considered as typical pathology of Alzheimer, is

related to the reactivation of mitotic mechanisms (Delobel et al., 2002). Another pathway we identified, named 'Progesterone-mediated oocyte maturation', is also involved in this process. It provides evidence that the pathway 'Oocyte meiosis' may be associated with BD. Hence, it is worthy to further study the relationship between oocyte meiosis and BD.

Another two remarkable pathways were 'Retinol metabolism' and 'Metabolism of xenobiotics by cytochrome P450'. The pathway 'Retinol metabolism' has been reported in a PBA study for BD (Smith et al., 2009), providing a validation for our result. 'Metabolism of xenobiotics by cytochrome P450' has not been reported in any BD PBA studies so far, but many studies that focused on the drug metabolism and treatment of BD have mentioned cytochrome P450. Quetiapine, which was found to be effective in the treatment of BD, is predominantly metabolized by cytochrome P450 3A4 (Khazaal et al., 2013).

References

Altar, C.A., Jurata, L.W., Charles, V., Lemire, A., Liu, P., Bukhman, Y., Young, T.A., Bullard, J., Yokoe, H., Webster, M.J., *et al.* (2005). Deficient hippocampal neuron expression of proteasome, ubiquitin, and mitochondrial genes in multiple schizophrenia cohorts. Biol Psychiatry 58, 85-96.

Bousman, C.A., Chana, G., Glatt, S.J., Chandler, S.D., Lucero, G.R., Tatro, E., May, T., Lohr, J.B., Kremen, W.S., Tsuang, M.T., *et al.* (2010). Preliminary evidence of ubiquitin proteasome system dysregulation in schizophrenia and bipolar disorder: convergent pathway analysis findings from two independent samples. Am J Med Genet B Neuropsychiatr Genet 153B, 494-502.

Chen, L., Zhang, L., Zhao, Y., Xu, L., Shang, Y., Wang, Q., Li, W., Wang, H., and Li, X. (2009). Prioritizing risk pathways: a novel association approach to searching for disease pathways fusing SNPs and pathways. Bioinformatics 25, 237-242.

Delobel, P., Flament, S., Hamdane, M., Mailliot, C., Sambo, A.V., Begard, S., Sergeant, N., Delacourte, A., Vilain, J.P., and Buee, L. (2002). Abnormal Tau phosphorylation of the Alzheimer-type also occurs during mitosis. J Neurochem 83, 412-420.

Donohue, T.M., Jr. (2002). The ubiquitin-proteasome system and its role in ethanol-induced disorders. Addict Biol 7, 15-28.

Hegde, A.N., and Upadhya, S.C. (2011). Role of ubiquitin-proteasome-mediated proteolysis in nervous system disease. Biochim Biophys Acta 1809, 128-140.

Huo, L.J., Fan, H.Y., Zhong, Z.S., Chen, D.Y., Schatten, H., and Sun, Q.Y. (2004). Ubiquitin-proteasome pathway modulates mouse oocyte meiotic maturation and fertilization via regulation of MAPK cascade and cyclin B1 degradation. Mech Dev 121, 1275-1287.

Khazaal, Y., Preisig, M., Chatton, A., Kaufmann, N., Bilancioni, R., and Eap, C.B. (2013). Use of high doses of

quetiapine in bipolar disorder episodes are not linked to high activity of cytochrome P4503A4 and/or cytochrome P4502D6. Psychiatr Q 84, 329-335.

Lam, Y.A., Pickart, C.M., Alban, A., Landon, M., Jamieson, C., Ramage, R., Mayer, R.J., and Layfield, R. (2000). Inhibition of the ubiquitin-proteasome system in Alzheimer's disease. Proc Natl Acad Sci U S A 97, 9902-9906.

Middleton, F.A., Mirnics, K., Pierri, J.N., Lewis, D.A., and Levitt, P. (2002). Gene expression profiling reveals alterations of specific metabolic pathways in schizophrenia. J Neurosci 22, 2718-2729.

O'Dushlaine, C., Kenny, E., Heron, E.A., Segurado, R., Gill, M., Morris, D.W., and Corvin, A. (2009). The SNP ratio test: pathway analysis of genome-wide association datasets. Bioinformatics 25, 2762-2763.

Ogata, H., Goto, S., Sato, K., Fujibuchi, W., Bono, H., and Kanehisa, M. (1999). KEGG: Kyoto Encyclopedia of Genes and Genomes. Nucleic Acids Res 27, 29-34.

Peng, G., Luo, L., Siu, H., Zhu, Y., Hu, P., Hong, S., Zhao, J., Zhou, X., Reveille, J.D., Jin, L., *et al.* (2010). Gene and pathway-based second-wave analysis of genome-wide association studies. Eur J Hum Genet 18, 111-117.

Price, A.L., Patterson, N.J., Plenge, R.M., Weinblatt, M.E., Shadick, N.A., and Reich, D. (2006). Principal components analysis corrects for stratification in genome-wide association studies. Nat Genet 38, 904-909.

Purcell, S., Neale, B., Todd-Brown, K., Thomas, L., Ferreira, M.A., Bender, D., Maller, J., Sklar, P., de Bakker, P.I., Daly, M.J., *et al.* (2007). PLINK: a tool set for whole-genome association and population-based linkage analyses. Am J Hum Genet 81, 559-575.

Ripke, S., Sanders, A.R., Kendler, K.S., Levinson, D.F., Sklar, P., Holmans, P.A., Lin, D.Y., Duan, J., Ophoff, R.A., Andreassen, O.A., *et al.* (2011). Genome-wide association study identifies five new schizophrenia loci. Nat Genet 43, 969-976.

Rubio, M.D., Wood, K., Haroutunian, V., and Meador-Woodruff, J.H. (2013). Dysfunction of the ubiquitin proteasome and ubiquitin-like systems in schizophrenia. Neuropsychopharmacology 38, 1910-1920.

Shimura, H., Schlossmacher, M.G., Hattori, N., Frosch, M.P., Trockenbacher, A., Schneider, R., Mizuno, Y., Kosik, K.S., and Selkoe, D.J. (2001). Ubiquitination of a new form of alpha-synuclein by parkin from human brain: implications for Parkinson's disease. Science 293, 263-269.

Smith, E.N., Bloss, C.S., Badner, J.A., Barrett, T., Belmonte, P.L., Berrettini, W., Byerley, W., Coryell, W., Craig, D., Edenberg, H.J., *et al.* (2009). Genome-wide association study of bipolar disorder in European American and African American individuals. Mol Psychiatry 14, 755-763.

Subramanian, A., Tamayo, P., Mootha, V.K., Mukherjee, S., Ebert, B.L., Gillette, M.A., Paulovich, A., Pomeroy, S.L., Golub, T.R., Lander, E.S., *et al.* (2005). Gene set enrichment analysis: a knowledge-based approach for interpreting genome-wide expression profiles. Proc Natl Acad Sci U S A 102, 15545-15550.

Wang, K., Li, M., and Bucan, M. (2007). Pathway-based approaches for analysis of genomewide association studies. Am J Hum Genet 81, 1278-1283.

Zhang, K., Chang, S., Guo, L., and Wang, J. (2014). I-GSEA4GWAS v2: a web server for functional analysis of SNPs in trait-associated pathways identified from genome-wide association study. Protein Cell.
